# The dengue-specific immune response and antibody identification with machine learning

**DOI:** 10.1038/s41541-023-00788-7

**Published:** 2024-01-20

**Authors:** Eriberto Noel Natali, Alexander Horst, Patrick Meier, Victor Greiff, Mario Nuvolone, Lmar Marie Babrak, Katja Fink, Enkelejda Miho

**Affiliations:** 1 FHNW University of Applied Sciences and Arts Northwestern Switzerland, School of Life Sciences, Muttenz, Switzerland; 2 Department of Immunology, Oslo University Hospital Rikshospitalet and University of Oslo, Oslo, Norway; 3 Department of Molecular Medicine, University of Pavia, Pavia, Italy; 4 ImmunoScape, Singapore, Singapore; 5 SIB Swiss Institute of Bioinformatics, Lausanne, Switzerland; 6 aiNET GmbH, Basel, Switzerland

**Keywords:** Adaptive immunity, Infection, Viral infection, Antibodies

## Abstract

Dengue virus poses a serious threat to global health and there is no specific therapeutic for it. Broadly neutralizing antibodies recognizing all serotypes may be an effective treatment. High-throughput adaptive immune receptor repertoire sequencing (AIRR-seq) and bioinformatic analysis enable in-depth understanding of the B-cell immune response. Here, we investigate the dengue antibody response with these technologies and apply machine learning to identify rare and underrepresented broadly neutralizing antibody sequences. Dengue immunization elicited the following signatures on the antibody repertoire: (i) an increase of CDR3 and germline gene diversity; (ii) a change in the antibody repertoire architecture by eliciting power-law network distributions and CDR3 enrichment in polar amino acids; (iii) an increase in the expression of JNK/Fos transcription factors and ribosomal proteins. Furthermore, we demonstrate the applicability of computational methods and machine learning to AIRR-seq datasets for neutralizing antibody candidate sequence identification. Antibody expression and functional assays have validated the obtained results.

## Introduction

Dengue (DENV) is a virus that poses a serious threat to global health as the etiological agent of dengue fever. Four distinct serotypes have been reported (DENV-1 to -4) which differ in the main immunodominant antigen Protein E (Envelope)^1^. One approved vaccine is the CYD-TDV^2^. However, while this can prevent the potentially fatal dengue hemorrhagic fever in previously infected individuals, it is ineffective in naïve (seronegative) individuals where it instead increases the risk of severe disease. Despite several development efforts, no DENV-specific therapeutics have been approved^3–5^. Neutralizing antibodies targeting the four serotypes represent a promising therapeutic for dengue^6^. While a few broadly reactive DENV-specific antibodies have been described (Table 1), only one has entered clinical development. Diverse broadly neutralizing antibodies (bNAbs) that can enter clinical development are needed to find an effective therapeutic for this unmet medical need.

**Table 1:** Dengue-specific bNAbs.

Antibody name	Type	Reference
9F12	bNAb	(Rajamanonmani et al.)^44^
3E31	bNAb	(Li et al.)^85^
M366.6	bNAb	(Midgley et al.)^45^
1A1-D2	bNAb	(Lok et al.)^46^
M360.6	bNAb	(Midgley et al.)^45^
2H12	bNAb	(Midgley et al.)^45^
4E11	bNAb	(Thullier et al.)^86^
Ab513	bNAb	(Robinson et al.)^9^
SIgN-3C	bNAb	(Xu et al.)^47^
2A10G6	bNAb	(Deng et al.)^87^
1C19	bNAb	(Smith et al.)^88^
d448	bNAb	(Li et al.)^89^
J8	bNAb	(Durham et al.)^90^
J9	bNAb	(Durham et al.)^90^
EDE1 C10	bNAb	(Rouvinski et al.)^29^
EDE1 C8	bNAb	(Rouvinski et al.)^29^
EDE2 A11	bNAb	(Rouvinski et al.)^29^
EDE2 B7	bNAb	(Rouvinski et al.)^29^
D23-1B3B9	bNAb	(Injampa et al.)^91^
DVD-1A1D-2A10	bNAb	(Injampa et al.)^91^
1F4	bNAb	(Shi et al.)^92^
2D22	bNAb	(Teoh et al.)^93^
1M7	bNAb	(Smith et al.)^88^
1N5	bNAb	(Smith et al.)^88^
4E5A	bNAb	(Thullier et al.)^86^
EDE bNAbs	Dataset of 50 bNAbs	(Dejnirattisai et al.)^30^

High-throughput sequencing of antibody repertoires has emerged in the last two decades as a promising tool for deep analysis of the immune response. This technology evolved parallel to advances in computational analyses to resolve the immune repertoire complexity and to understand the dynamics of adaptive immunity^7–9^. Selected types of computational analytics, such as the network theory^10^, biological diversity indexes^11^, statistical analysis of germlines and complementarity determining region 3 (CDR3), and mathematical modeling of VDJ recombination of antibodies^12^ have been employed to investigate antibody repertoires. These approaches offer a unique opportunity to study the antibody response to dengue and elucidate the immunological fingerprint elicited by the different DENV serotypes on the repertoire. Machine learning methods can be applied to understand the specificity of antibody sequences at a single-cell level and their developability potential as therapeutics^6,13–15^. Moreover, single-cell RNA sequencing (scRNA-seq) technology enables the understanding of cellular responses by assessing gene expression levels at the single-cell resolution^16^. These approaches have been extensively specifically leveraged to generate AIRR sequencing data and study the adaptive immune receptor repertoire (AIRR).

**Fig. 1:**
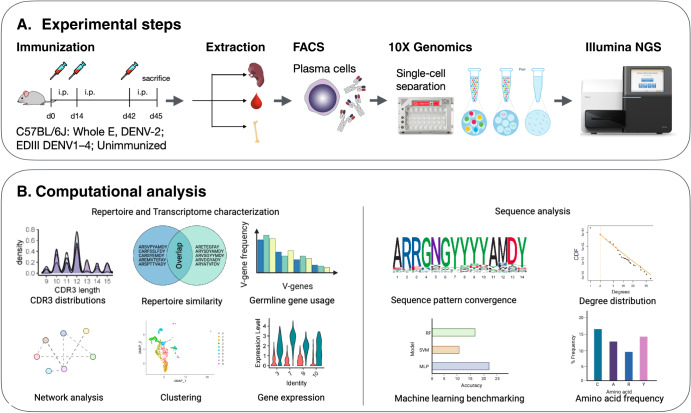
Experimental design and analysis. **a** Experimental steps. Five cohorts of three mice each were immunized with dengue antigens Whole protein E DENV-2 (mice 4, 5, 6), EDIII from DENV-1 (mice 9, 10, 11), EDIII from DENV-2 (mice 12, 13, 14), EDIII from DENV-3 (mice 15, 16, 17), EDIII from DENV-4 (mice 18, 19, 20), while one cohort of three mice was treated with adjuvant only (mice 1, 2, 3). Mice were sacrificed and bone marrow was collected. Plasma cells were FACS-sorted. Antibody repertoires were sequenced, and high-throughput sequencing datasets were annotated and preprocessed. **b** Computational analysis performed. On the left, repertoire-level: CDR3 overlaps (repertoire similarity), germline gene usage, uniform manifold approximation and projection (UMAP) clustering, network analysis, CDR3 length distributions, gene expression. On the right, sequence-level: sequence conservation, an example of clone similarity degree distribution of an antibody repertoire, machine learning benchmarking, amino acid frequency.

Here, we set out to examine the immune response to the different DENV serotypes at the antibody repertoire and sequence level (Fig. 1). Moreover, we implemented and optimized an antibody discovery workflow integrating machine learning to identify broadly neutralizing antibody candidates. We used this method to identify DENV-specific B-cell clones from sorted bone marrow plasma cells of mice immunized with DENV-1, DENV-2, DENV-3 and DENV-4 antigens of varying complexity, thus enhancing screening to fewer, less time-consuming and less expensive steps. Furthermore, we investigated the transcriptional effects of DENV and identified genes with an altered expression in dengue infection. Our findings provide insights into the humoral response to the dengue virus and pipelines for the routine application of computational approaches to identify potential broadly neutralizing antibodies. This workflow has the potential to be applied to other diseases that elicit a specific antibody response.

## Results

We have characterized the antibody repertoire in response to immunization with dengue virus antigens of increasing complexity such as the domain III of protein E of each DENV1-4 serotype (EDIII-1, −2, −3, −4) and the whole protein E of DENV2 including all domains I, II and III (Whole E-2). We sorted long-lived plasma B cells from bone marrow, and used high-throughput single-cell sequencing to obtain 47.8 million raw reads. In order to understand the adaptive immune response to dengue and identify antibody sequences with dengue-specific binding potential, we analyzed global sequence-space and transcriptome characteristics of the repertoires, and we detected patterns at the repertoire and a.a. sequence level.

### Dengue antigen immunization increases the diversity of the antibody repertoire

Infectious diseases leave a fingerprint on the immune repertoire^17^. We focused on the immunological fingerprint left on the bone marrow, as this is the niche containing the long-lasting plasma cells responsible for life-long immunity^18^. With the aim to characterize the long-lived plasma cells arising in mice from dengue antigen immunization in this compartment, we selected two fundamental parameters: V/J germline genes, essential for the development of effective immune responses^19,20^ and heavy chain CDR3, the principal determinant of antibody specificity and therefore a hallmark for the repertoire binding capability^21^. These parameters were analyzed to characterize the different signature left by the DENV virus serotypes on the repertoire and by antigens of increasing structural complexity, both on a repertoire level and a sequence level.

**Fig. 2:**
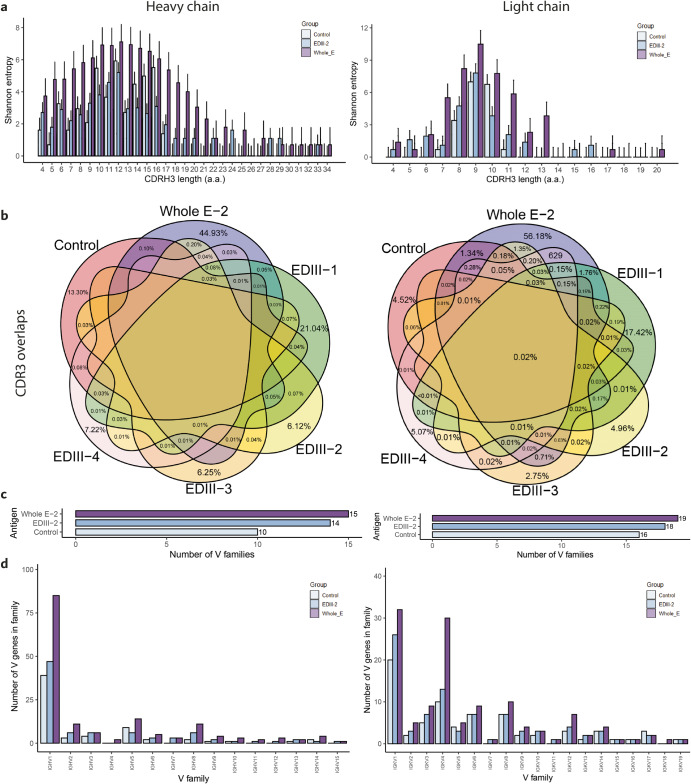
Dengue antigen immunization imprints on the antibody repertoire an immune signature of increased diversity. **a** Shannon entropy values per CDR3 length of control (light blue bars), EDIII-2 (dark blue bars) and Whole E-2 (violet bars) immunized mice for heavy (left) and light (right) chains. Bars represent standard error. **b** Percentage of shared heavy (left) and light (right) chain CDR3 between control, Whole E and EDIII-1, EDIII-2, EDIII-3 and EDIII-4 immunized mice. **c** Total number of V gene families present in heavy (left) and light (right) chain repertoires of control, EDIII-2 and Whole E-2 immunized mice. **d** Number of subtype V genes per V-gene family in heavy (left) and light chain (right) repertoires.

To compare how the repertoire diversity changes when immunization occurs with two antigens of increasing complexity, we calculated the Shannon entropy of the length of heavy and light chain CDR3s in cohorts immunized with EDIII-2, Whole E-2, and control cohorts (Fig. 2). For CDR3 lengths between 4 and 17 amino acids, the entropy of the control and the EDIII-immunized mice presented comparable levels, while the entropy of the Whole E cohort was greater than any of these two cohorts for each CDR3 value. For CDR3 containing >17 amino acids the entropy of the control cohort was zero, while the two DENV-immunized cohorts showed positive entropy values. The Whole E-2 cohort presented positive entropy for all CDR3 lengths from 4 to 34 amino acids. In case of the light chain repertoires, the Whole E-2 mice had CDR3 with greater entropy than control and EDIII for CDR3 lengths between 4 and 13 amino acids. For CDR3 >13 amino acids, the control showed positive entropy for 15 and 16 amino acids, and Whole E showed positive entropy for 17 and 20 amino acids. Our results show that dengue antigen immunization is correlated with an increase in the entropy of CDR3 lengths, and this entropy increases with the complexity of the dengue antigen. Furthermore, to reconfirm that the increase of repertoire diversity is unique to the dengue antigens (Fig. 2a) and does not occur upon immunization with other antigens, we compared the Shannon diversity per CDR3 length in mice immunized with additional antigens OVA and hepatitis B Virus antigen (HbsAg) (Supplementary Fig. 3b). Immunization of mice with OVA or HbsAg caused a decrease in repertoire diversity reflected by a lower Shannon entropy across CDR3 length. In terms of percentage of overlapping CDR3 (Fig. 2b), both in case of heavy and light chain CDR3s, the mice immunized with Whole E-2 presented the greatest percentage of CDR3 exclusively present in the cohort (44.93% for the heavy chains and 56.18% for the light chains). The control and the EDIII-immunized cohorts showed a lower percentage of cohort-exclusive CDR3, ranging from 6.12% (EDIII-2) to 21.04% (control) for the heavy chains and 2.75% (EDIII-3) to 17.42% (EDIII-1) for the light chains. These results indicate that dengue Whole E-2 complex antigen leaves a more individualized signature in the adaptive immune response and that the increase of diversity is limited to dengue and not typical of other antigens.

Next, we analyzed and compared germline gene usage in control, EDIII-2 and Whole E-2 mice. For both heavy and light chains, the mice immunized with Whole E-2 showed repertoires retaining the largest number of V genes (Fig. 2c). Whole E had 15 heavy chain V genes and 19 light chain V genes, EDIII had 15 heavy chain V genes and 18 light chain V genes, and the control contained 10 heavy chain V genes and 16 light chain V genes. Moreover, for IGHV1, IGHV2, IGHV4, IGHV5, IGHV6, IGHV8, IGHV9, IGHV10, IGHV11, IGHV12, IGHV14, IGHV15 and IGKV1-6, IGKV8-9, IGKV12, IGKV14 mice immunized with Whole E-2 had a greater number of V genes per family (Fig. 2d). Collectively these results showed that when mice are immunized with dengue antigens, the diversity of the antibody repertoire of the long-lived plasma cells in the bone marrow increases by causing a more varied usage of V gene families, and also V genes within each family, rather than causing a usage of fewer V families. This effect is more pronounced when the Whole protein E, the more complex antigen, is used for the immunization, rather than the less complex antigen EDIII.

### Dengue antigen immunization elicits a signature of long CDR3 rich in polar amino acids

**Fig. 3:**
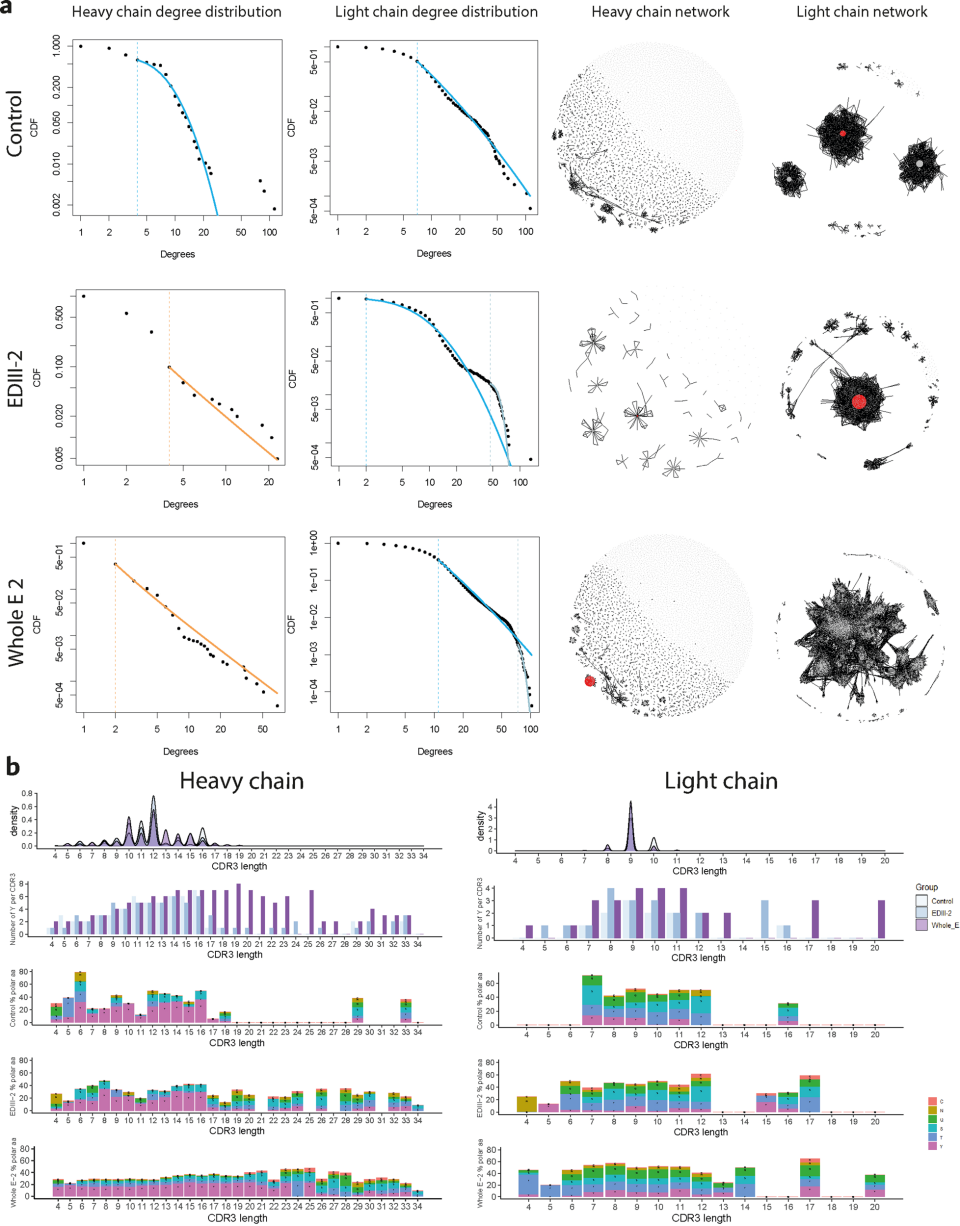
Dengue elicits a clonal expansion and somatic hypermutation inducing a power-law-like degree distribution on the repertoire, with development of long CDR3 sequences rich in polar amino acids. **a** Left panel: degree distributions of control, EDIII-2 and Whole E-2 immunized exemplary mice in each cohort for heavy and light chains; lognormal distributions lines are in blue, Poisson distributions lines are in gray, power-law distributions are in yellow. Right panel: the respective graphical representation of the networks. The top frequency clone of each repertoire is colored in red and the other clone CDR3s are colored in gray. **b** Top to bottom: CDR3 length plotted against CDR3 frequency, Tyrosine counts (Y), polar amino acids C, N, Q, S, T, Y sequence percentage in the CDR3 for BMPC datasets from control, EDIII-2 and Whole E-2 mice. Panels on the left refer to heavy chain data, panels on the right to light chain data.

Previous application of the network theory to the antibody repertoires has shown that antigen-exposure events such as infection or vaccination can change the architecture of antibody repertoires by causing selective expansion and somatic hypermutation of certain clones, thus inducing network structures focused on certain high-degree clones with a power-law distribution^10^. Moreover, it has been observed that antibodies targeting glycosylated antigens, e.g., viral antigens such as HIV gp120, present long heavy chain CDR3 which allow them to protrude between the glycan shields and reach the respective epitope^22^. Furthermore, amino acids presenting polar side chains are key players in protein-protein interactions involving glycans^23,24^. To characterize the DENV heavy and light chain antibody repertoire, we applied network theory to the repertoire of long-lived plasma cells in the bone marrow of mice immunized with EDIII from DENV-2 and Whole E DENV-2 (Fig. 3b). Initially, we calculated degree distributions, which provide an immediate indication of how similarities (degrees) between antibody sequences are distributed in the repertoire. The control mouse repertoires presented a degree distribution with many clones having a low degree (i.e., many CDR3s which had a low number of similar CDR3), for the heavy and the light chains, indicating greater diversity of the repertoire. Mice immunized with protein EDIII-2 and Whole E-2 showed a power-law-like degree distribution for the heavy chain repertoire, and for the light chain repertoire a mixed degree distribution where the clones with lower degree followed a lognormal distribution and the ones with the higher degree a Poisson distribution. Subsequently, we applied the network theory to visualize heavy and light chain repertoires as networks in which each CDR3 is represented by a node and the CDR3 which differ by 1 amino acid are connected by a similarity edge (black dashed line). The network is particularly efficacious at representing the repertoire architecture qualification and has enabled detection of somatic hypermutation in antibody-related diseases such as leukemia^25^ and HIV^26^. The networks of control mice heavy and light chain repertoires showed 3 big clusters of many interconnected nodes and low degree clones, and other clusters with a tree-like structure (54 for the heavy chains and in 59 for the light chains). The EDIII mice showed for the heavy chains a network with 8 star-like structures and for the light chain one central, predominant cluster containing one high-degree clone. For Whole E-immunized repertoires, the heavy chain showed the presence of one high degree clone, and the light chain one central cluster made of 22478 interconnected clones with low degree. From these results it can be inferred that dengue antigens of increasing complexity impacts repertoire sequence diversity architecture. The heavy chain repertoire architecture reflected polarization onto certain predominant clones, switching from a degree distribution with many clones with low degree in the control, to a power-law degree distribution for EDIII-2 and Whole E-2. When visualizing the networks as graphs we observed organization in star-like structures which are compatible with clonal expansion, and presence of one clone with high degree in Whole E-2 mice. In terms of light chains, one possible explanation for the mixed lognormal-Poisson distribution of EDIII and Whole E could be the presence of multiple light chains pairing with single heavy chains.

Subsequently, we evaluated the physicochemical composition of the repertoire and CDR3 length (Fig. 3b). As a hallmark of glycan-binding capability, considering that dengue protein E is glycosylated, we characterized the percentage of polar amino acids Cysteine (C), Asparagine (N), Glutamine (Q), Serine (S), Threonine (T) and Tyrosine (Y)^27^ and the number of Y in the CDR3 sequences, and compared these parameters in control mice, EDIII-2 and Whole E-2 immunized mice for heavy and light chains. The reason for selecting also Y counts alone as a parameter lies in the fact that it has already been established that the amino acid Y plays a crucial role in the paratope of DENV broadly neutralizing antibody CDR3s which bind to certain epitope structures on dengue protein E through Y-repeated motifs such as YYY or YYYY^28–30^. Control, EDIII and Whole E had similar CDR3 length frequency distributions, with most of the CDR3s having a length between 4 and 19 amino acids for the heavy chains and between 7 and 11 amino acids for the light chains. Moreover, for heavy chain CDR3s long 4 to 16 amino acids, the Y counts and polar amino acid percentage per CDR3 were similar among the control, EDIII-2 and Whole E-2 groups. When analyzing longer heavy chain CDR3 (≥17 amino acids), the control was highly different from the two DENV-immunized datasets: EDIII and Whole E developed long CDR3 with high Y counts and enrichment (higher frequency) in the polar amino acids C, N, Q, S, T, Y. With regards to the light chains, CDR3 longer than 11 amino acids showed no Y except for the length of 16 amino acids in the control, while EDIII presented Tyrosines in CDR3 long 12, 15 and 16 amino acids and Whole E presented Tyrosines in 12, 13, 17, and 20 amino acids long CDR3s. Interestingly, the maximum number of Y per heavy chain CDR3 present in Whole E cohort (*n* = 8) was double than the one for the light chains (*n* = 4), suggesting that the composition of Y in the heavy chain CDR3 is more relevant for dengue antigen binding with respect to the light chain CDR3. Control light chain CDR3 longer that 11 amino acids contained polar amino acids only for a length of 16 amino acids while EDIII contained polar amino acids in lengths 12, 15, 16, 17 amino acids and Whole E in more lengths, specifically 12, 13, 14, 17, 20 amino acids. We observed that mice immunized with other antigens such as OVA or HbsAg did not develop antibodies with long CDR3s (i.e., >28 a.a., Supplementary Fig. 3a) and the CDR3 lengths distribution was largely overlapping between the control and OVA-/HbsAg-immunized mice, within the ranges between 4 a.a. and 20 a.a. of length. There was no enrichment in polar amino acids in the repertoire of control, OVA- and HbsAg-immunized mice (Supplementary Fig. 3c–e) compared to DENV (Fig. 3b). Collectively, dengue protein immunization imprints on the antibody repertoire a signature with longer CDR3 sequences which are rich in polar amino acids, especially for the heavy chain and, to a minor extent, for the light chains. These longer CDR3 sequences might enable simultaneous binding of anti-DENV antibodies with polar amino acids to epitopes on the envelope protein E glycans and to other epitopes on the amino acid backbone of protein E.

### Bone marrow plasma cells after immunization with dengue protein E domain III present higher levels of expression of transcription factor subunits and ribosomal proteins

**Fig. 4:**
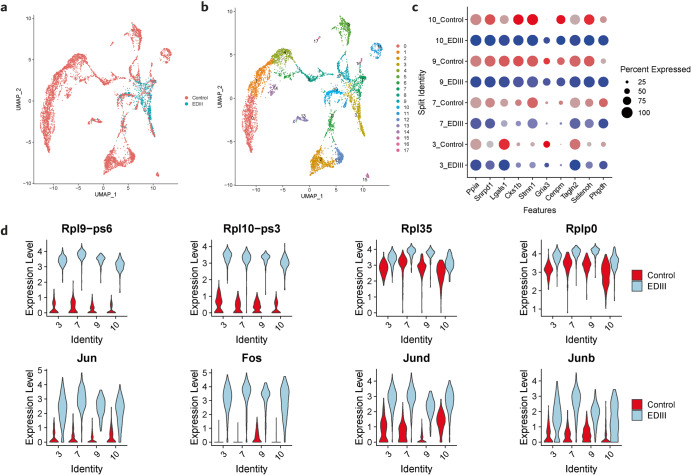
Dengue elicits an antibody expression-enhanced state in bone marrow plasma cells. **a** Uniform manifold approximation and projection (UMAP) of transcriptomics in control and EDIII mice; cells of the control are in red, cells of EDIII are in light blue. **b** UMAP clustering of the integrated BMPC control and EDIII with respect to 18 clusters (0-18); (**c**) Gene expression of top 10 differentially expressed genes in overlapping clusters 3, 7, 9, 10. Genes from control are indicated in red, EDIII genes are in light blue. **d** Gene expression of ribosomal protein gene expression (top 4) and transcription factors (bottom 4) in overlapping clusters 3, 7, 9, 10. Violin plots for the control are in red, violin plots for EDIII are in light blue.

Infection with dengue virus causes changes in the transcription level of various genes (Supplementary Table 2). These include interferon genes involved in the antiviral responses, inflammatory processes control, transcription factors, immune cell development regulation, energetic processes and other genes involved in various cellular processes^31–33^. Previous studies have focused on the investigation of transcriptional effects of dengue on activated plasmablasts in peripheral blood during the acute phase. Whether dengue antigen immunization imprints transcriptional changes in the long-lived plasma cells in the bone marrow has not been elucidated yet. To address the transcriptional diversity of long-lived plasma cells in the bone marrow after serial immunization with EDIII, we performed a comparative analysis of the transcriptome of control and EDIII DENV-2-immunized mice. Anchor cells in biologically matched transcriptional state (Fig. 4a) were preprocessed in order to remove outlier cells (see Methods and Supplementary Fig. 2). Seventeen transcriptional clusters were identified in the integrated dataset by employing principal component analysis to analyze differentially expressed genes (Fig. 4a). Further analysis was performed on clusters 3, 7, 9 and 10 because these clusters contained the matched long-lived plasma cells in the bone marrow. These clusters showed comparable levels of transcription of genes which are normally expressed in bone marrow plasma cells^34–37^ (Supplementary Fig. 4), enabling antiviral responses and regulation of inflammatory mechanisms, such as interferon genes and genes controlling inflammatory mechanism pathways. Moreover, CD47 and metabolic genes had comparable levels of expression. The top 10 differentially expressed genes showed a similar percentage of expression in all clusters except for the gene Gria3 (Fig. 4c) which was differentially expressed only in cluster 10 but showed similar expression in all the other clusters. Genes that showed a significant different expression between the control and the EDIII cells encoded for ribosomal proteins (Rpl9-ps6, Rpl10-ps3, Rpl35, Rplp0) and transcription factors of the JNK (Jun, Jund, Junb) pathway and the Fos family. These genes were expressed at a higher level in the dengue sample (Fig. 4d). Results were statistically significant, with *p*-values lower than 10^−22^ (Supplementary Table 3). JNK are proteins of the kinases family which can be activated by different triggers^38^ and the Jun proteins, key players of it, can dimerize with genes of the Fos family thereby forming transcription factor complex AP-1^38^ which triggers various responses including transcription of ribosomal protein genes^39–41^. These results suggest that activation of naïve B cells with dengue antigens poses cells under a transcriptional state which enhances protein expression, likely to potentiate differentiation and antibody expression, which is maintained after the terminally differentiated long-lived plasma cells reach the bone marrow.

### Dengue viral infection imprints the adaptive immune response with long CDR3 and Y-rich motifs based on IGHJ6 usage in human memory B cell repertoires

**Fig. 5:**
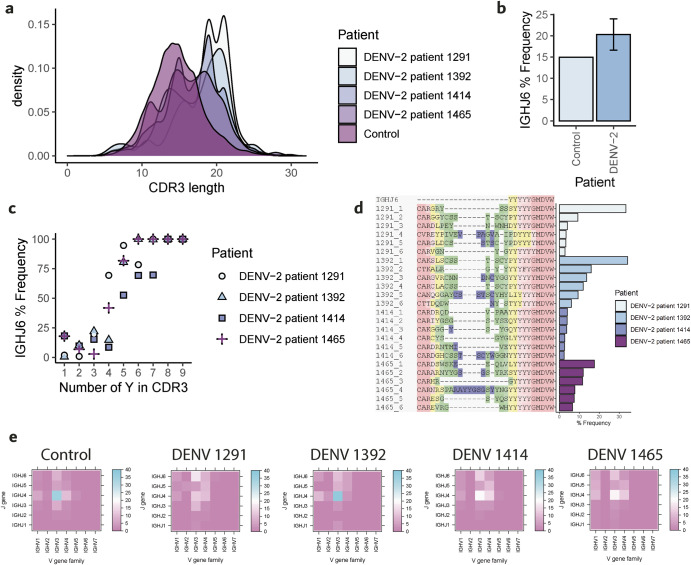
Dengue imprints long CDR3 and Y-rich motifs thanks to IGHJ6 usage of the memory B-cell repertoires. **a** CDR3 length of control and DENV-2 patient memory B cells (white = dengue patient 1291; light blue = dengue patient 1392; light purple = dengue patient 1414; purple = dengue patient 1465; dark purple = control patient). **b** Frequency of IGHJ6 usage in memory B-cell repertoires of control (light blue) and DENV-2 (dark blue) samples (bar represents sample variance). **c** Frequency of IGHJ6 usage per number of CDR3 Tyrosines (Y) in dengue patient samples 1291 (dots), 1392 (triangles), 1414 (squares), 1465 (crosses). **d** Alignment of IGHJ6 CDR3 sequence with the top 6 frequency memory B cell clones in dengue patients 1291 (1291_1 to 1291_6), 1392 (1392_1 to 1392_6), 1414 (1414_1 to 1414_6), 1465 (1465_1 to 1465_6) and relative frequency in each repertoire. **e** Percentage frequency of V–J family gene pairs in control and dengue patient samples.

The activation of memory B cells (MBC) contributes to a substantial part in the antibody response to dengue, resulting in an increase of neutralizing antibody titers, having a key role in the immunological response to the virus^42^. To characterize the immunological fingerprint produced by dengue on the human MBC antibody repertoire, we analyzed MBC antibody repertoires of four patients affected by acute DENV-2 (namely patient 1291, 1392, 1414, 1465) from the study by Appanna and coworkers^43^ (Fig. 5). Patients affected by DENV-2 were selected because of the widely reported capability of this DENV serotype to elicit bNAbs^29,44–47^. As we previously observed in mice bone marrow long-lived plasma cells data, dengue elicited antibodies with long CDR3s in the MBC repertoire of dengue patients^43^ shown also in their distributions (Fig. 5a). The frequency of the usage of the germline gene IGHJ6 was greater in all DENV patients than in the control (Fig. 5b), suggesting that IGHJ6 may play a key role in the immune response to the virus. When further investigating the role of IGHJ6, we observed that the increase of usage of this J gene corresponded to increased counts of Tyrosine amino acids in the CDR3 (Fig. 5c), to the point that antibodies containing 9 Y in the CDR3 derived only from the IGHJ6 gene. A sequence alignment of the part of the IGHJ6 sequence that recombines into the CDR3 (YYYYYGMDV) with the top 6 frequency memory B cell clones in the four DENV patients showed that all clones ended in the terminal part of the CDR3 with a motif of repeated Tyrosines (from 4 to 6). This Y-motif was followed by sequences of amino acids such as GMDVW or MDVW generally from IGHJ6, underlining that it is the recombination of the IGHJ6 with other V and D germline genes that enables the formation of high frequency clones (in some cases, >30% of the repertoire) containing repeated Tyrosine amino acids. The IGHJ6 paired preferentially with the V gene family IGHV3 in the DENV MBC patient samples, but also in Control. Moreover, in DENV patients IGHJ6 paired with IGHV1 with a higher frequency than in the control cohort. Collectively, the human MBC data analyzed in Appana et al. and here reflects our observations regarding murine bone marrow plasma cells data. Dengue elicits long CDR3s and Y-rich motifs in the antibody repertoire. These features may provide MBC a prompt activation in the case of a dengue viral infection and allow a strong, broadly neutralizing response through antibodies that can bind to glycans and other epitopes on the E protein. In addition, we elucidated that the mechanism at the base of the development of the Y-rich CDR3 is the usage of the IGHJ6, which contains repeated Tyrosine amino acids.

### Benchmarking of machine learning algorithms for paired heavy and light chain sequence classification and computational identification of dengue broadly neutralizing antibody clone candidates

**Fig. 6:**
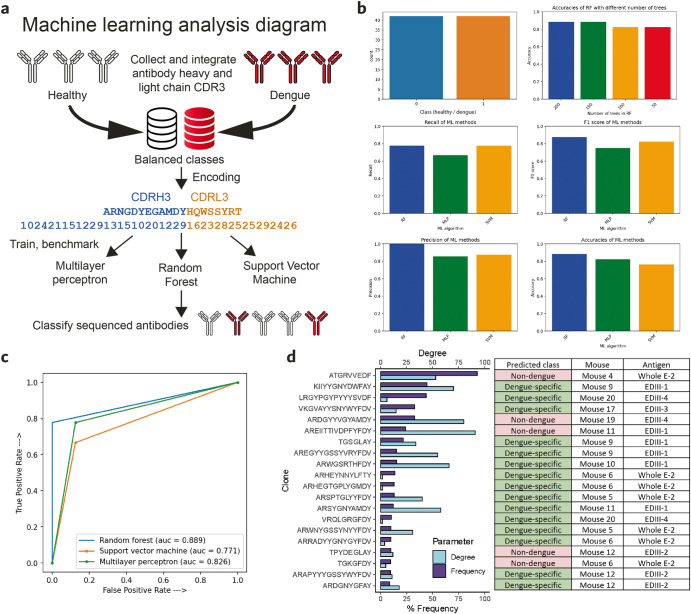
Benchmarking of different machine learning algorithms for paired heavy-light chain antibody sequence classification and identification of potential dengue neutralizing antibody binders. **a** Graphical representation of the computational pipeline. **b** From top left to bottom right: number of paired heavy and light chain CDR3 sequences in training datasets; accuracy of random forest algorithm trained with 200, 150, 100 and 50 decision trees; precision, recall, F1 score and accuracy comparison between random forest (RF) with 200 trees, support vector machines (SVM) and multilayer perceptron (MLP). **c** Receiver operating curve (ROC) of random forest with 200 trees, support vector machine and multilayer perceptron. **d** Top identified 20 clones in dengue datasets, predicted class (non-dengue or dengue-specific), mouse eliciting the clone and antigen.

Previous studies have demonstrated the capability of machine learning models to identify intrinsic patterns of sequencing data and predict antigen specificity in antibody sequences^13,48,49^. Prediction of specificity has been limited only to the heavy chain sequence. One reason is because retaining paired heavy chain–light chain data from bulk B-cell populations at the single-cell level has been a challenge for a long time^50^, leading to a lack of paired AIRR heavy and light chain sequencing datasets. The light chain plays a key role not only in binding to the antigen, but also in the positioning of the CDR3 of the heavy chain^51^. Hence, the need to incorporate the light chain CDR3 as a feature for more reliable and robust prediction of antibody specificity. In order to achieve classification of paired heavy and light chain antibody sequences, we extended the encoded features to the light chain and benchmarked different machine learning algorithms for predicting paired heavy and light chain CDR3 data. Moreover, we combined various computational approaches through complementing machine learning sequence classification with statistical analyses and network theory^10^ in order to identify the top 20 binding clone candidates of the sequenced datasets (Fig. 6). Firstly, we collected paired heavy and light chain antibody sequencing data from healthy patients from a previous study by Goldstein and coworkers^52^ and integrated it with the paired heavy and light chain CDR3 sequences of dengue broadly neutralizing antibodies^13^, achieving a machine learning training dataset with balanced classes for dengue-specific and non-dengue sequences (Fig. 6a). The paired heavy and light chain CDR3 sequences of the training dataset were integer-encoded where each amino acid was represented as an integer (10–29) and fed for training into three machine learning models for sequence classification. Random forests, multilayer perceptron and support vector machines were used based on their suitability for this classification task as previously described^13^. A Random forest model was trained with different numbers of trees (50, 100, 150, 200) and benchmarked for accuracy, with 150 and 200 trees resulting in the highest prediction accuracies. Once selected the random forest with 200 trees, it was compared in terms of accuracy, precision, recall and F1 score (Fig. 6b) as well as ROC curve (Fig. 6c) with the other two models. The best performing model was random forests, which was therefore selected for classifying clones in the dengue datasets (Fig. 6d). Of 20 selected sequence clones, 15 were classified as “dengue-specific” and 5 as “non-dengue.” The clones identified were named as “DenAb” followed by a different letter for each clone (e.g., DenAb A, DenAb B etc.). These results show that machine learning reconfirmed most of the top clones identified through proprietary analytics being classified as “dengue-specific.” The putative DENV-specific antibody clone DenAb X was expressed and tested for binding to DENV Whole E antigens by ELISA to confirm antigen specificity of the identified clones, resulting in the antibody binding to DENV-1, DENV-2 and DENV-3 serotypes (Supplementary Fig. 5). The computational identification of these antibody paired heavy and light sequence clones paves the way for their further expression and laboratory validation.

## Discussion

Dengue poses a serious threat to global health causing a potentially fatal hemorrhagic fever or dengue shock syndrome. Passive delivery of an efficacious broadly neutralizing antibody would represent a prophylactic and therapeutic solution for the treatment to this devastating disease. Several dengue bNAbs have been reported (Table 1), but only one of these is in clinical trials. Moreover, the only available vaccine is inefficacious at protecting naïve individuals from the infection and disease. Next generation sequencing and computational analytics have enormously evolved in the last decade and provide a unique opportunity to deconvolute the dengue immune response and identify potential therapeutics for dengue. Here we applied different computational approaches to elucidate the immune response to dengue, both in mice immunized with viral antigens of increasing complexity and of different dengue serotypes (whole protein E from serotype 2 and EDIII from serotypes 1 to 4), and in patients affected by acute dengue.

We observed that immunization with dengue proteins increases the diversity of the antibody repertoire in the long-lived plasma cells in the bone marrow compartment both at a CDR3 and a germline gene usage level. It is known that viral antigens (e.g., with vaccination or infection) cause changes in repertoire diversity. However, when the antibody repertoire is presented with an antigen, it typically decreases in diversity because of the shift towards usage of certain V-genes that allow the generation of highly potent, antigen-specific antibodies, for example in influenza^53^, SARS-CoV2^54^, hepatitis C^55^, vesicular stomatitis virus (VSV)^56^. In the case of dengue, instead, one previous study reports an increase in B-cell repertoire diversity in peripheral blood of patients with acute phase disease^57^, imputing it to the massive mobilization and expansion of different dengue-specific plasmablast clones that occur in dengue^58^. We observe an increase of diversity following dengue antigen immunization in another compartment, the bone marrow. This suggests that the increase of diversity may not be due to plasmablast mobilization, but to other factors. One recent study by Hyatt and coworkers^59^ showed that dengue EDIII changes conformation when in contact with certain glycosaminoglycans expressed on host cells, like Heparin and Chondroitin sulfate C. We reconfirmed that repertoire diversity increase in the long-lived bone marrow plasma cells was specific to dengue and did not occur for other antigens like hepatitis B virus antigen or ovalbumin. While this was observed for the heavy chain CDR3, one limitation is that we did not have light chain data for the mice immunized with these other OVA and HbsAg, but considering that the heavy chain CDR3 is the most relevant region for antigen-binding, if present, an increase of diversity should have likely shown up in this region. One possible explanation for the increase in repertoire diversity is that these structural changes of EDIII may give rise to a mixed antigen population which presents diversified epitopes to the immune system and therefore the antibody repertoire diversifies to adapt to the different antigen structures. This hypothesis could be confirmed by further structural studies.

In agreement with previous studies^28^, we observed that dengue elicited the development of antibodies with a heavy chain CDR3 containing repeated Tyrosines “YYYY” sequence motifs, both in mice BMPC as recombinant antigen and in human memory B cells when the live virus is causing an infection. These motifs constitute a signature of dengue infection on the antibody repertoire; one structural explanation for their development is that they constitute paratopes through which bNAbs can bind to the E protein^29,30^ and recognize different serotypes. We observed that Tyrosine was not the only polar amino acid amplified in the CDR3 regions upon dengue antigen immunization, but also other polar amino acids (C, N, Q, S, T) represented an increasing portion in long CDR3s. It is established that viral antigens such HIV elicit long heavy-chain CDR3 which allow these antibodies to reach the cognate epitope protruding through the heavy glycan shields of the protein gp120. The effect of dengue antigen immunization might, in an analogous way, trigger development of antibodies that with long CDR3 and Y-rich motifs can bind simultaneously to glycans and other amino acid residues on the backbone of the dengue E protein. Moreover, we showed that the mechanism on which the development of Y-rich CDR3s occurs is the frequent usage of the IGHJ6 gene which contains repeated Tyrosine amino acids. It is known that DENV infection does not elicit many neutralizing antibodies targeting EDIII^60^, and that potent DENV neutralizing antibodies rather recognize complex epitopes formed by E dimers^30^ or whole virus^61^. In this study, we observed the development of global repertoire signatures common between mice immunized with recombinant antigen and humans infected with live virus such as greater diversity, longer CDR3 and CDR3 rich in polar amino acids. These global features are more likely a consequence of the development of cross-reactive, non-neutralizing antibodies, which are most commonly triggered by DENV, than due to the rare and single neutralizing antibodies which are less common and underrepresented in the repertoire. Hence, our study further underlines the need for computational strategies to identify the rare neutralizing DENV antibodies from within antibody repertoires.

We applied the network theory to antibody heavy and light chain repertoires, and calculated degree distributions as this method is particularly efficacious when searching for repertoire architecture features^10,25,26^. Previous work by Miho and coworkers has shown that the long-lived plasma cell compartment of B-cell repertoires in mice grown in experimental setting without immunization reflects an exponential degree distribution, as a result of the presence of many diverse B-cell clones which are not expanded or somatically hypermutated^10^. This is what we observed in the control heavy and light chain samples. When an antibody repertoire is immunized with an antigen, certain antigen-specific clones expand and somatically hypermutate, resulting in a pool of clones that have a small difference one to another (e.g., LD = 1 or 1 amino acid of difference in the CDR3). Indeed, we observed that when mice were antigen-immunized with dengue serotype 2 the repertoire architecture mutated to a power-law distribution and a network structure with star-like clusters containing more similar clones to a central clone. The light chain degrees showed a mixed lognormal-Poisson distribution. We limited our analysis to LD = 1 similarity networks, however further analyses to additional similarity layers are needed (e.g., LD ≥ 2) to investigate more in depth the architecture of the antibody repertoire in dengue^10^.

It is yet unknown whether dengue infection can change the transcriptional status of long-lived plasma cells in the bone marrow. Previous studies of the transcriptional effect of dengue on B cells have established that it regulates the expression of a series of genes which are correlated to antiviral responses, inflammatory processes, transcription factors, immune cell development regulation, energetic processes and other genes involved in various cellular processes^31–33,62,63^. We investigated the influence of viral antigen immunization on the transcription of genes in the long-lived plasma cells in the bone marrow because these cell population has a key role in regulating immunological processes. Dengue EDIII enhanced the expression of ribosomal protein genes and transcriptional factors of the Jun and Fos pathways. These are key elements of a pathway that starts with the dimerization of Jun and Fos genes, which form a transcription factor complex that triggers transcription of ribosomal protein genes. Therefore, we hypothesize that dengue may put the activated naïve B cells in an enhanced transcriptional state that promotes expression of antibodies, once the terminally differentiated long-lived plasma cells reach the bone marrow. One limitation of our approach is that we used Freund’s adjuvant for immunizing mice, which contains mycobacteria and therefore could skew the response to an antibacterial inflammatory response, promoting enhanced transcription of factors of the Jun and Fos pathways which are known for being proinflammatory cascades against bacteria^64,65^. Further immunization experiments with recombinant protein without adjuvant or live virus infection would be needed to reconfirm that this effect observed in the bone marrow plasma cells is not an effect due to the adjuvant. Moreover, additional experiments to investigate the transcriptional effect on long-lived plasma cells of other control antigens are needed to fully establish that the observed effect is unique to DENV. One additional limitation is that in this study we limited our analysis only to the serotype 2 and we didn’t study the transcriptional effects of DENV1, 3 and 4, but the previous work by Zanini et al.^31^ has shown that the transcriptional effects of DENV on PBMC in different patients affected by different serotypes is similar. Additional experiments with the other serotypes would be needed to confirm that this holds true for the bone marrow long-lived plasma cells. The challenge of understanding the mechanism with which the dengue antigens can activate the transcription of these factors, which are at the top of the transcriptional cascade, might be solved through interactomics in the future.

Identification of broadly neutralizing antibody clones has been historically a long, laboratory-intensive process in which collections of recombinant antibody libraries or B-cell secreted antibodies must be tested for binding to an antigen^66^ before identifying lead candidates. Given the enormous diversity of the B-cell repertoire, only few clones usually bind to the desired antigen and a vastity of other clones are excluded in the processes. Conversely, our antibody discovery approach starts from deep sequencing of the antibody repertoire and implements computational tools which allow selection of top candidates before expressing the antibodies and testing them, narrowing down the binding screening steps to only a few top clones. To mine the obtained datasets searching for possible dengue broadly neutralizing antibody candidate clones, we combined various computational tools: (i) a statistical approach^67^, (ii) the network theory^10^ and (iii) machine learning methods^13^. Models were trained with paired heavy and light chain CDR3 sequence data in order to achieve a complete characterization of the heavy and light chain. The paired information provides an improved representation of the antibody binding capabilities, and is an addition with respect to previous models based only on single heavy chain CDR3 data^49^. The top performing machine learning model was implemented to classify antibody sequences based on their CDR3. Through the computational approaches, we identified 20 clones, 15 of which were classified as “dengue-specific” by machine learning models. We expressed one of the putative anti-DENV antibodies (DenAb X) to confirm the accuracy and specificity of the pipeline. When tested for binding, the antibody was able to bind to the DENV serotypes 1, 2 and 3. This preliminary result shows that the identified antibody could bind to the DENV antigen protein E and paves the way for further testing of this antibody (e.g., binding against DENV-4, affinity testing, neutralization) and expression and testing of the other identified antibodies. This work demonstrates the applicability of computational methods and machine learning to high-throughput antibody repertoire sequencing datasets to identify broadly neutralizing antibodies. Furthermore, we envision computational methods to become in the near future a standard tool for rapid and machine learning-driven identification of antigen-specific high-affinity binding antibody sequences and instructing developability, thus closing the gap between selected antibodies and effective therapeutics.

## Methods

### Datasets

Dataset 1: the NGS dataset was generated in-house. It contains high-throughput single-cell sequenced samples of plasma cells extracted from the bone marrow of 18 mice. The experimental pipeline for its generation is described in Methods.

### Experimental pipeline

Dataset 2: the dataset from Parameswaran et al.^28^ was generated performing twice sequencing of heavy-chain IgGs by independent GS FLX (454 Life Sciences/Roche) runs in 60 individuals: 44 patients from Nicaragua, of which 8 were healthy individuals, and 44 patients with dengue infection in different phases: acute, persistent after clearance of infection (convalescent), baseline profiles within the same post-covalescent individual (post-covalescent), and 8 patients without dengue. The data was downloaded from BioProject ID PRJNA205206.

Dataset 3: the dataset from Godoy-Lozano et al.^57^ was generated by sequencing the IgG heavy chain repertoires with Roche 454 from 19 patients in Mexico. Samples were taken during acute dengue and, for 11 patients, during post-convalescence. Quality filter was passed for raw sequences with an average ≥ Q28 value and reads ≥ 250 bp. The data was downloaded from BioProject ID PRJNA302665.

Dataset 4: the dataset from Huang et al.^68^ was generated by sequencing the heavy chain IgG and IgA repertoires on an Illumina NextSeq machine at 150 bp paired-end reads of 14 patients including patients with dengue, hemorrhagic dengue and healthy samples in Taiwan. Reads included had a Phred quality score of ≥ 33. The data was downloaded from BioProject ID PRJEB13768.

Dataset 5: the dataset from Appanna et al.^43^ was generated by sequencing the heavy chain variable regions through Roche 454 at 400 bp single-ends reads from 12 dengue patients in Singapore.

Dataset 6: the dataset is a database of heavy chain sequences of 27 broadly neutralizing antibodies that were able to recognize at least three different dengue serotypes and their epitopes recognized different domains of the E protein. The database was constructed based on literature search.

Dataset 7: the dataset includes ~62 million sequences from healthy patients collected from the public repository iReceptor^69^. Prior to download, the selection criteria were set accordingly to obtain only heavy chain productive sequences. Sequences were annotated with IMGT/V-QUEST^70^. To make the sequences annotated with IMGT comparable to the other datasets’ ones annotated with IgBLAST, leading cysteine (C) and trailing tryptophan (W) amino acids were removed from IMGT-blast sequences to match the IgBLAST output^71^. The dataset from Vander Heiden et al.^72^ was used a*s* healthy control for human memory B cells.

### Experimental pipeline

Mice immunizations were performed with the following protocol to generate Dataset 1. We immunized C57BL/6 J mice with dengue recombinant antigens. Immunization was carried out at the Pharmaseed animal facility in Ness-Ziona (Israel), housing a vivarium with a surgery suite and several procedure rooms. Animals were housed under standard laboratory conditions in individual ventilated cages. Handling was performed according to guidelines of the National Institute of Health (NIH) and the Association for Assessment and Accreditation of Laboratory Animal Care (AAALAC). This study was performed in compliance with “The Israel Animal Welfare Act” and following “The Israel Board for Animal Experiments” Ethics Committee approval # IL-20-1-22. A total of 18 male C57BL/6 J mice were divided into six cohorts, each containing three animals (Table 2). The animals were allocated into 5 immunization cohorts (total 15 mice) and 1 control cohort (3 mice, unimmunized). The immunization cohorts received an intradermal injection of Whole Protein E from dengue serotype 2 (Native Antigen Company, Oxfordshire, UK) (cohort 1) or Protein E domain III (cohort 2, 3, 4 and 5 injected with DENV-1, -2, -3 or -4 respectively) on Day 1, with boosters on Day 14 and Day 37. On Day 1, antigens were prepared in Complete Freund’s Adjuvant, on Days 14 in Incomplete Freund’s adjuvant and on day 37 in PBS buffer. The vehicle (CFA or IFA) was always at concentration 0.1 mg/mL. Mice injected with Whole E received 29 µg antigen/mouse in 0.2 mL vehicle. Mice treated with EDIII received 50 µg antigen/mouse in 0.1 mL vehicle. Control mice received 0.1 mL Complete Freund’s Adjuvant on Day 1, Incomplete Freund’s adjuvant on Day 14 and PBS on day 37 without any antigen. Mice were sacrificed on day 42. Bone marrow samples were harvested. Long-lived plasma cells were FACS-sorted with markers CD138 + CD22–MHCII–CD19–IgM–live/dead^73^. Two additional mice were used for bleeding for baseline serum production for ELISA. For the investigation of repertoire features present in mice immunized with additional antigens, ovalbumin (OVA) and hepatitis Virus B antigen (HbsAg), bone marrow plasma cell heavy chain high-throughput sequences from cohorts of three mice each were analyzed. Data was generated as previously described by Greiff et. al.^74^. Briefly, three cohorts of C57BL/6 J mice (Janvier Laboratories, France) 8–10 weeks old were intraperitoneally injected with alum-precipitated antigen (100 mg OVA, Invivogen), 4 mg HbsAg (Cell Sciences) in PBS with booster with identical quantity of antigen after 3 weeks. One cohort of mice was untreated. Fourteen days post-secondary immunization, mice were sacrificed and bone marrow cells were collected. Plasma cells were isolated with FACS.

**Table 2:** Mice cohorts, antigens, doses, and route of administration in this study.

Cohort number	Mouse number	Treatment	Dose level of the antigen (µg/mouse)	Dose volume (mL/mouse)	Route of administration
**1**	1,2,3	CFA/IFA/PBS only	0	0.1	intradermal
**2**	4,5,6	Antigen 1 (Whole E, DENV-2) in CFA/IFA/PBS	29 µg/mouse	0.2	
**3**	9,10,11	Antigen 2 (EDIII, DENV-1) in CFA/IFA/PBS	50 µg/mouse	0.1	
**4**	12,13,14	Antigen 3 (EDIII, DENV-2) in CFA/IFA/PBS	50 µg/mouse		
**5**	15,16,17	Antigen 4 (EDIII, DENV-3) in CFA/IFA/PBS	50 µg/mouse		
**6**	18,19,20	Antigen 5 (EDIII, DENV-4) in CFA/IFA/PBS	50 µg/mouse		

During the process of immunization, ELISA of the sera extracted from mice was performed (Supplementary Fig. 1). The purpose of this ELISA was to ascertain that the mice elicited an immune response against the antigens. ELISA plates were coated with 100 μL antigen at 1 μg/mL (overnight), blocked with 150 μL 1% BSA (60–90 min); 100 μL of each sample were added (60–90 min), detected with 100 μL HRP conjugated anti-mouse IgG secondary antibody (60 min). Substrate was then added. Serum dilution factor was determined by a preliminary calibration. The sera were diluted 1:50,000.

Single B cells were isolated using the 10X Genomics Chromium Single Cell V(D)J platform v1 (protocol version M) for single-cell immune profiling of full-length antibody variable heavy and light chain (VDJ) libraries following manufacturer’s instructions. Cell samples were loaded onto the Chromium Controller and partitioned into nanoliter-scale Gel Beads-in-emulsion (GEMs). The mRNA was reverse transcribed in cDNA, with all generated cDNA within one partition sharing a common 10X Barcode into each GEM. The VDJ sequences were enriched with a two-step PCR. Sequences were then fragmented and indexed with indices compatible with Illumina sequencing. Quality control of the materials obtained throughout the process and library concentration quantification was performed using an Agilent Bioanalyzer with suitable kit (Agilent BioAnalyzer High Sensitivity Kit). Antibody library pools at concentration 8pM were sequenced with Illumina MiSeq platform at 2 × 300 bp paired-end reads using the MiSeq Reagent kit v3 (600 Cycles). Mean base-call quality of all samples was in the range of Phred score 30.

Transcriptomics libraries were prepared according to the 10X Genomics Chromium Single Cell V(D)J protocol for single-cell immune profiling starting from cDNA samples. Briefly, the cDNA was enzymatically fragmented and adapters for illumina sequencing were added. Libraries were loaded onto an illumina NextSeq 550 instrument at 1.8 pM concentration using NextSeq 500/550 High Output Kit v2.5 (300 Cycles). Sequencing was performed at paired end 2 × 150 bp reads. Mean base-call quality of all samples was in the range of Phred score 30.

The putative DENV-specific antibody clone DenAb X was expressed to confirm antigen-specificity for DENV. The sequences of the heavy and of the light chain of the antibody were ordered from GeneArt (Thermo Fisher GeneArt, Regensburg, Germany) pre-cloned into the pcDNA 3.4 TOPO mammalian expression vectors. The monoclonal antibody was expressed in HEK (Human Embryonic Kidney) cells using the Expi293 expression system kit (Thermo Fisher, Carlsbad, CA, USA) following the instructions of the provider. Briefly, cells were cultured in 125-mL Erlenmeyer shaker flasks and incubated at 37 °C with a humidified atmosphere containing 8% CO2. The cell cultures were simultaneously transfected with heavy and light chain plasmids and the cell culture supernatant containing the expressed antibody was harvested 7 days post-transfection. After clarification of the supernatant, the antibody was purified with protein G spin columns with the Cytiva Protein G HP Ab SpinTrap kit (Cytiva, Freiburg im Breisgau, Germany). SDS-PAGE was performed to assess purity.

For testing binding of DenAb X to DENV, ELISA Maxisorp plates (Nunc, Rochester, United States) were coated with DENV Whole protein E from serotypes DENV-1, DENV-2 and DENV-3 produced by the Native Antigen Company (Native Antigen Company, Oxfordshire, UK). The coating was performed at 4°C overnight with 100 µl of antigen 10 µg/ml. The tested antibody was added at 10 µg/ml and binding was detected by adding a secondary anti-mouse HRP-conjugated antibody (Jackson Immunoresearch, West Grove, United States) diluted 1:5000, with TMB substrate used for the color reaction. Plates absorbances were read on a Flexstation 3 (VWR, Pennsylvania, United States).

### Computational pipeline

Dataset 1 raw sequences were demultiplexed with Cellranger mkfastq and subsequently V, D, J genes were annotated with Cellranger VDJ 5.0 and IgBLAST version 1.14. Datasets 2–5 were annotated with IgBLAST version 1.14.

Preprocessing was performed to retain only those clones that had CDR3 sequence length ≥4 and <35 amino acids and occurred more than once in each CDR3 repertoire dataset in order to filter out potential spurious results from sequencing errors. Only productive sequences were retained. Clones were defined by 100% amino acid sequence identity of CDR3^74^ and clonotypes by same CDR3 amino acid sequence, V and J gene^11^.

Raw reads were aligned to the mouse reference genome mm10 using Cellranger Count from the 10X Genomics software suite. The bone marrow tibia cell transcriptomics dataset generated by Perez et al. (SRA accession number SRX12344225 or Biosample accession number SAMN21618494) was used as control. Computational analysis was performed using the R Package Seurat^75^. Preprocessing of transcriptomics datasets was performed by removing outlier cells from the datasets (Supplementary Fig. 2), retaining only cells with number of features <5800 and number of RNA counts <45,000. Quality control was performed to check that no mitochondrial DNA was present in the analyzed samples. Data was integrated by finding the “anchors”, defined as cells in matched biological transcriptional state across different samples. Principal component analysis (PCA) clustering of the integrated dataset cells was performed implementing Uniform Manifold Approximation and Projection (UMAP). Statistical significance of the difference in gene expression was assessed by calculating *p*-values of the observed difference between the control and EDIII groups with Seurat.

For calculating the diversity of antibody repertoires of mice immunized with dengue antigens, we used the Shannon entropy index, with the rationale that this index is well established for measuring the diversity immune signature of antibody repertoires^11^. Briefly, we calculated the entropy for each given CDR3 length within reach repertoire based on Eq. 1.

1 $$H=-\mathop{\sum }\limits_{i=1}^{S}{p}_{i}{\log }_{b}{p}_{i}$$

where *p(i)* is the probability of outcome *i*, so the probability that one CDR3 of length *p* has a certain length *i* in a repertoire with S different CDR3 lengths. For example, a repertoire where there are 4 CDR3 with identical length of 10 a.a. each, will have an entropy of −(4/4 * log(4/4)) = 0, while a repertoire where there are 4 CDR3 of 4 different lengths like 10, 11, 12, 13 amino acids, the Shannon Entropy will be −(1/4 * log(1/4) + 1/4 * log(1/4) + 1/4 * log(1/4) + 1/4 * log(1/4)) = 1.39. The index was calculated for each *i* clonotype within all the lengths observed in our datasets (4 amino acids to 34 amino acids).

Antibody repertoires were represented as networks as previously described^10^, drawing from the network theory^75^ and translating the concepts of network analysis^76^. Briefly, repertoires were represented as undirected graphs G = (V, E) defined by a set of nodes (or vertices, V, which correspond to the CDR3 in the repertoire) and a set of connections (similarity edges, E), representing the adjacency matrix of pairwise Levenshtein distance equal to one (LD = 1, one amino acid difference between CDR3 amino acid sequences). For example, the CDR3 CARTA and CARRTA have distance LD = 1. In the context of antibody repertoires, we let *N* = |V| and *L* = |E|. The size of a graph N is the number of its CDR3 sequences (nodes). The degree k represents the number of edges connected to a node and describes the number of similar clones to one CDR3 which have LD of 1. The degree may be interpreted as a measure of how central a CDR3 clone is in the antibody repertoire sequence space. In simpler terms, it quantifies the number of CDR3 clones that are similar to a certain CDR3, and thus the potential development or the evolutionary routes to this CDR3. The centralization analysis indicates if the network is homogeneous (clones are connected in the same way) or is centered around certain nodes (highly connected clonal regions compared to less connected regions in the same network). The largest component is the largest cluster of connected CDR3 clones. The diameter (d) is the maximum distance (shortest path between two nodes) between any pair of CDR3 sequences.

Degree distributions and model fits were calculated for antibody repertoires. When network theory is applied to antibody repertoires, the degree of a CDR3 sequence represents the number of CDR3 sequences with a certain Levenshtein distance from it. For example, if one CDR3 has a degree of 3 in a similarity layer of LD = 1, there are 3 CDR3 in the repertoire that have LD = 1 from it. The degree distribution P(k) = Nk/N, defined as the fraction of nodes with degree k (Nk) in total nodes (N), represents the fraction of CDR3 clones that have the same number of similar CDR3s. The power-law, exponential and Poisson distributions were fitted to the empirical degree distributions of the repertoire networks, constructed as described in Network analysis, by estimating x_min_ (estimated lower degree threshold by minimizing the Kolmogorov-Smirnoff statistic) and optimizing model parameters using the poweRlaw package^76^ as previously described^10^.

The frequency of CDR3 clones was calculated as the count of the occurrences of each CDR3 divided by the sum of the counts of all clones within a sample. The frequency of V, D, and J-gene families was calculated as the count of the first V, D, and J-gene hit occurrence of each CDR3 clone divided by their sum within the sample.

Machine learning for paired heavy and light chain sequence classification and computational identification of dengue broadly neutralizing antibody clones was performed using Python 3.8.3^77^. Initially, a data set was created, containing a balanced number of paired heavy and light chain dengue sequences from publicly available data of 50 dengue bNAb HCDR3/LCDR3 sequences from Dejnirattisai et al.^30^ that were labeled as “dengue-specific”, and non-dengue antibody paired HCDR3/LCDR3 sequences from a study of paired VH/VL single cell sequencing dataset from healthy donors from Goldstein et al.^52^ that was labeled as “non-dengue”. These paired sequences were integer encoded in vectors where each amino acid is replaced by a number of two digits (e.g., *A* = 10, *C* = 11, *D* = 12, *E* = 13 etc). so a sequence “AADEC” will be encoded in the vector (1010121311). Considering that CDR3s had different lengths, the CDR3s which had a length lower than the highest one present in the dataset (for heavy chain: 26 a.a., for light chain: 14 a.a.) were padded at the end with a series of “30” to achieve vectors of the same length. The dataset was split in 80% training and 20% testing sets with 5-fold cross validation, and was used to train machine learning algorithms Random Forest, Support vector machine and a neural network (Multilayer Perceptron). The neural network consisted of two hidden layers with respectively 26 and 14 neurons, in order to reflect the maximum length of the HCDR3 of 26 a.a. and the LCDR3 of 14 a.a. In the dataset. We benchmarked the algorithms in terms of accuracy, precision, recall and F1 score with the Random Forest performing the best. The Random Forest was tested with different numbers of trees (50, 100, 150, 200) and we selected the one with 200 trees which had the greatest accuracy (0.889) for antibody sequence classification. Subsequently, we encoded the paired HCDR3/LCDR3 sequences from the antibodies that we identified with Network analysis with the same integer encoding method and used the Random Forest to classify these sequences as “dengue-specific” or “non-dengue.”

Statistical analysis of datasets was conducted using Rstudio 1.4.1106^78^ and Python 3.8.3^77^. All scripts are uploaded to GitLab under https://gitlab.fhnw.ch/aihealthlab/dengue-analytics and are available upon approval of research request. Graphics were generated using the R packages ggplot2^79^, Venn^80^, ggseqlogo^81^, corrplot^82^, pheatmap^83^, treemap^84^.

### Reporting summary

Further information on research design is available in the Nature Research Reporting Summary linked to this article.

## Supplementary information

Supplementary Information

REPORTING SUMMARY

## Data Availability

Raw data is available in Zenodo: https://zenodo.org/record/7414892#.Y6ApjnaZNaR. Code is available upon approval of research request.
