# Author Correction: The dengue-specific immune response and antibody identification with machine learning

**DOI:** 10.1038/s41541-024-00820-4

**Published:** 2024-02-13

**Authors:** Eriberto Noel Natali, Alexander Horst, Patrick Meier, Victor Greiff, Mario Nuvolone, Lmar Marie Babrak, Katja Fink, Enkelejda Miho

**Affiliations:** 1 FHNW University of Applied Sciences and Arts Northwestern Switzerland, School of Life Sciences, Muttenz, Switzerland; 2 Department of Immunology, Oslo University Hospital Rikshospitalet and University of Oslo, Oslo, Norway; 3 Department of Molecular Medicine, University of Pavia, Pavia, Italy; 4 ImmunoScape, Singapore, Singapore; 5 SIB Swiss Institute of Bioinformatics, Lausanne, Switzerland; 6 aiNET GmbH, Basel, Switzerland

**Keywords:** Adaptive immunity, Infection, Viral infection, Antibodies

Correction to: *npj Vaccines* 10.1038/s41541-023-00788-7, published online 20 January 2024

In this article, the affiliation details for author Alexander Horst were incorrectly given as Alexander Horst^1,2^ but should have been Alexander Horst^1^ and other affiliations are renumbered. The original article has been corrected.

