# Publisher Correction: The dengue-specific immune response and antibody identification with machine learning

**DOI:** 10.1038/s41541-024-01027-3

**Published:** 2024-12-09

**Authors:** Eriberto Noel Natali, Alexander Horst, Patrick Meier, Victor Greiff, Mario Nuvolone, Lmar Marie Babrak, Katja Fink, Enkelejda Miho

**Affiliations:** 1 FHNW University of Applied Sciences and Arts Northwestern Switzerland, School of Life Sciences, Muttenz, Switzerland; 2 Department of Immunology, Oslo University Hospital Rikshospitalet and University of Oslo, Oslo, Norway; 3 Department of Molecular Medicine, University of Pavia, Pavia, Italy; 4 ImmunoScape, Singapore, Singapore; 5 SIB Swiss Institute of Bioinformatics, Lausanne, Switzerland; 6 aiNET GmbH, Basel, Switzerland

**Keywords:** Adaptive immunity, Infection, Viral infection, Antibodies

Correction to: *npj Vaccines* 10.1038/s41541-023-00788-7, published online 20 January 2024

In the original version of this Article, the code availability conditions were not clear in the Methods section. The article has now been corrected to clarify that research requests for access to the code need to be approved by the code owners.

