# Supplementary material for: The dengue-specific immune response and antibody identification with machine learning
Source: NPJ Vaccines. 2024 Jan 20;9:16. doi: 10.1038/s41541-023-00788-7 (PMC10799860; doi:10.1038/s41541-023-00788-7)
Supplement: Supplementary file 1 — Supplementary Information [file 41541_2023_788_MOESM1_ESM.docx]

**Supplementary Information**

**The dengue-specific immune response and antibody identification with machine learning**

Eriberto Noel Natali^1^, Alexander Horst^2^, Patrick Meier^1^, Victor Greiff^3^, Mario Nuvolone^4^, Lmar Marie Babrak^1^, Katja Fink^5^, Enkelejda Miho^1,6,7,*^

^1^FHNW University of Applied Sciences and Arts Northwestern Switzerland, School of Life Sciences, Muttenz, Switzerland

^2^Swissmedic, Basel, Switzerland

^3^Department of Immunology, Oslo University Hospital Rikshospitalet and University of Oslo, Norway

^4^Department of Molecular Medicine, University of Pavia, Pavia, Italy

^5^ImmunoScape, Singapore

^6^SIB Swiss Institute of Bioinformatics, Lausanne, Switzerland

^7^aiNET GmbH, Basel, Switzerland

*Corresponding author: Prof. Dr. Enkelejda Miho, enkelejda.miho@fhnw.ch

**SUPPLEMENTARY INFORMATION**

**Supplementary figures**

**
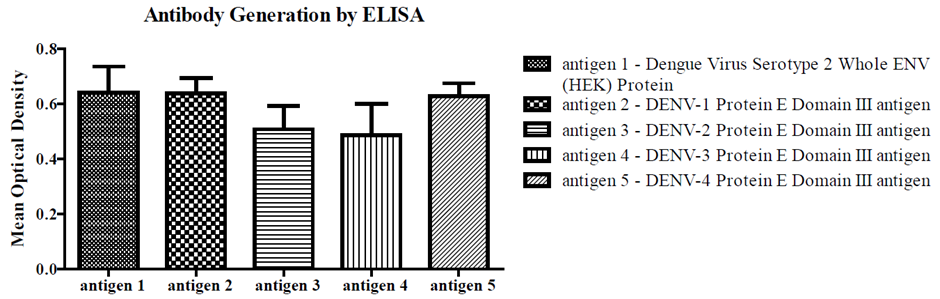
**

Supplementary Figure 1. Evaluation of Antibody generation via ELISA in the five cohorts immunized with different dengue antigens. Data are presented as mean across the three mice of each immunization cohort + SEM.


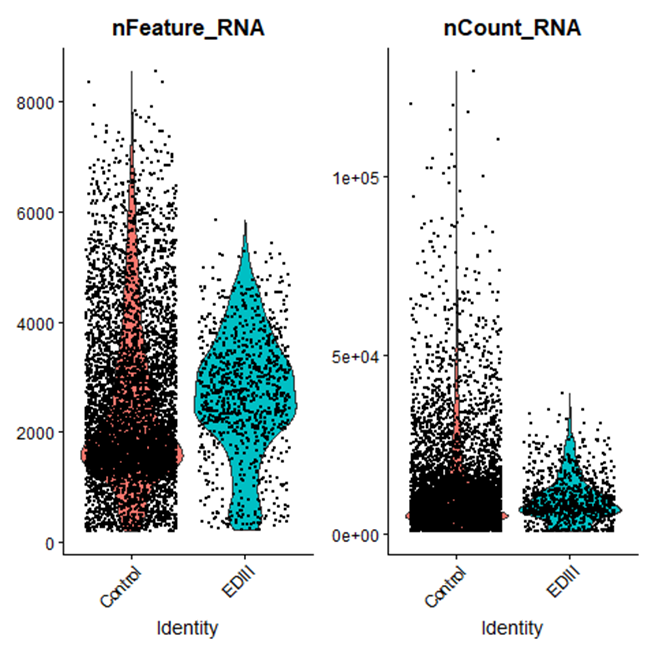


Supplementary Figure 2. Preprocessing of DENV transcriptomics datasets. Left: number of features (genes) per cell in control and EDIII datasets. Right: number of RNA molecules per cell in control and EDIII datasets. The control violin plot is in red, the EDIII violin plot is in blue. Each cell is represented by a black dot.

Supplementary Figure 3. Repertoire features of bone marrow plasma cells in mice immunized with OVA and Hepatitis B virus antigen. **a** CDR3 length if Control, OVA- and HbsAg-immunized mice (purple = HbsAg, green = control, yellow = OVA). **b** Shannon entropy values per CDR3 length of control (green bars), OVA (yellow bars) and HbsAg. **c** Polar amino acids C, N, Q, S, T, Y sequence percentage in the CDR3 for BMPC datasets from control mice. **d** Polar amino acids C, N, Q, S, T, Y sequence percentage in the CDR3 for BMPC datasets from OVA-immunized mice. **e** Polar amino acids C, N, Q, S, T, Y sequence percentage in the CDR3 for BMPC datasets from HbsAg-immunized mice. CDR3 length, Shannon Entropy and percentage of polar amino acids were calculated among datasets of 3 mice per each antigen.
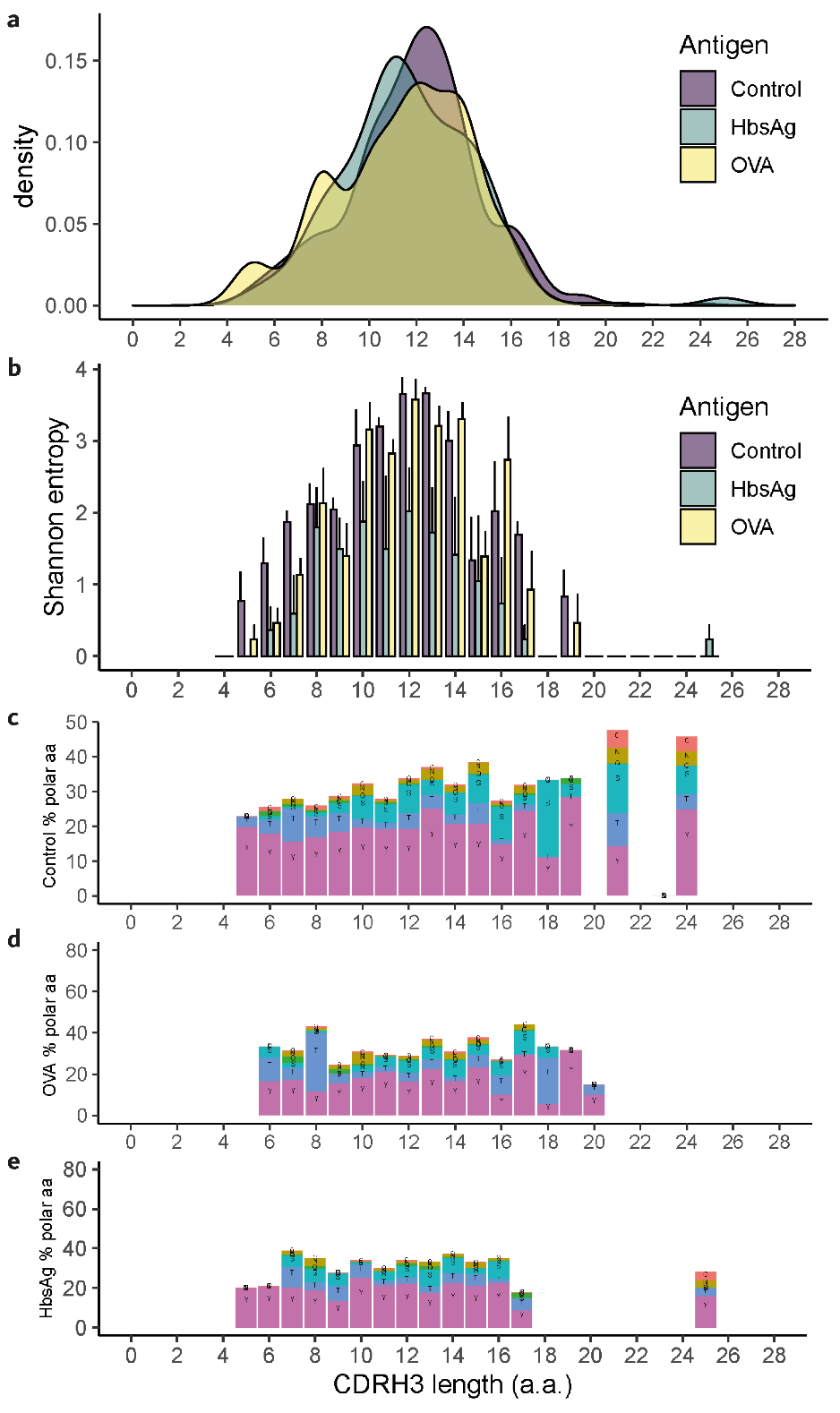


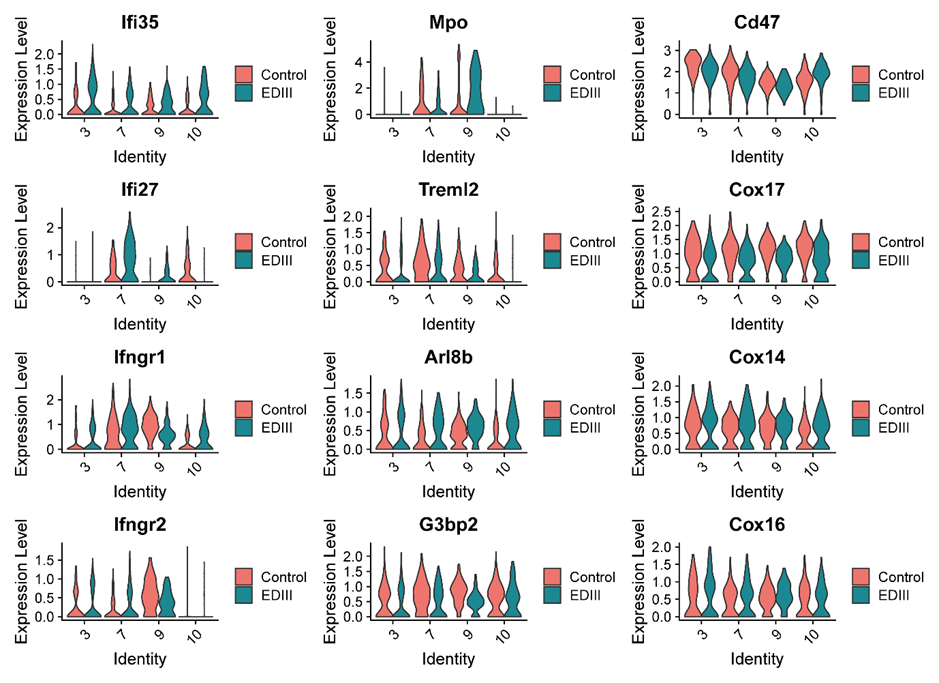


Supplementary Figure 4. comparative analysis of gene expression of interferon genes (first column), inflammatory pathway control genes (second column), other genes (third column), between the control cell sample and the sample mouse 12 BMPC (immunized with EDIII).


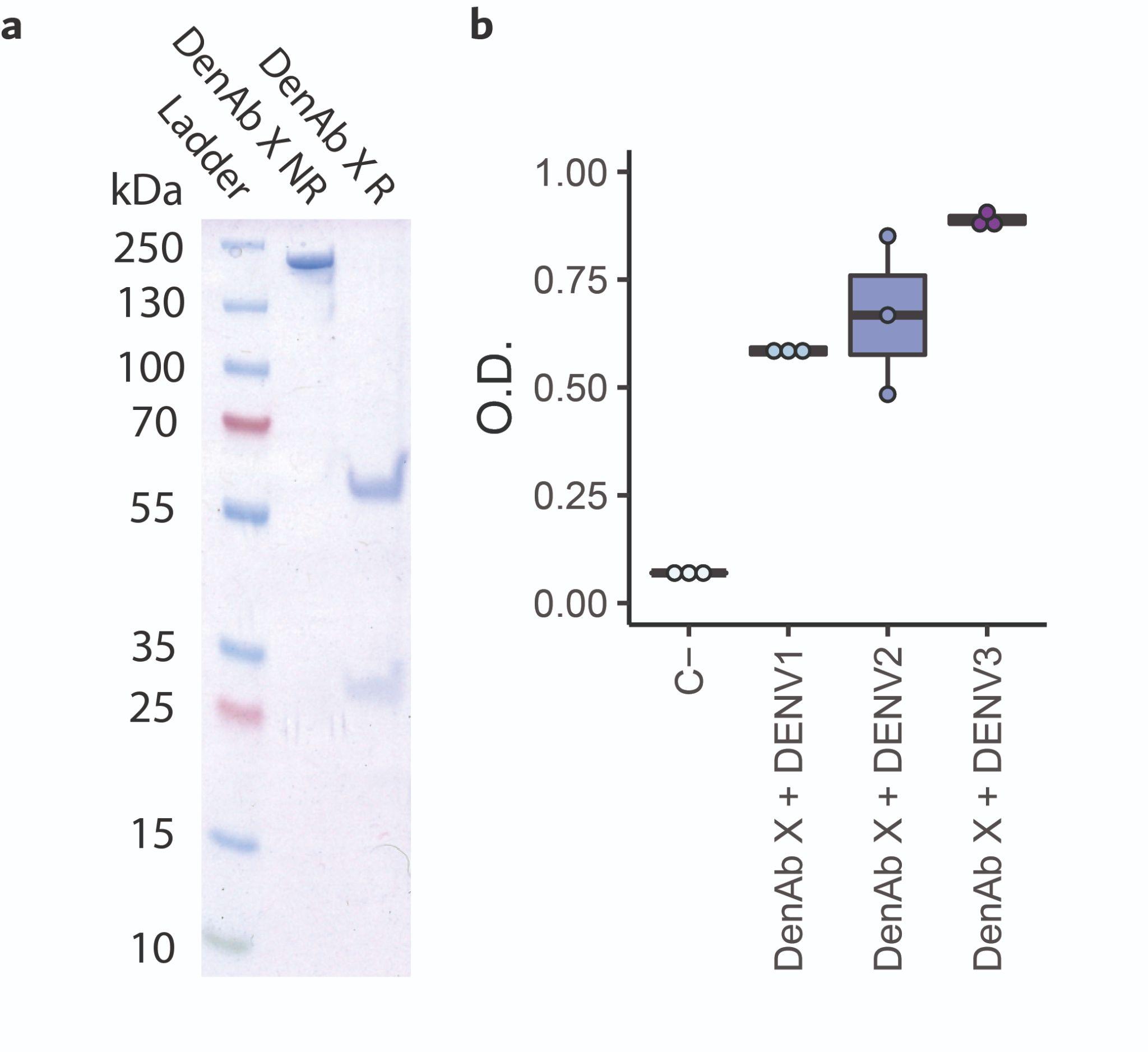


Supplementary Figure 5. Expression and binding of DenAb X to DENV. **a** SDS-PAGE of purified DenAb X with the antibody ran under non-reducing (DenAb X NR) or reducing (DenAb X R) conditions. The reducing agent added in the “R” well is dithiothreitol (DTT) which causes a separation of the heavy chains from the light chains, causing the appearance of two bands instead of one. **b** ELISA binding of the antibody to DENV-1, DENV-2 and DENV-3 Whole E DENV protein. Dots correspond to different measurements of the O.D. (absorbance at 450nm) in the same experiment (C- = negative control, DenAb X + DENV1 = binding of DenAb X to Whole E DENV1; DenAb X + DENV2 = binding of DenAb X to Whole E DENV2; DenAb X + DENV3 = binding of DenAb X to Whole E DENV3).

**Supplementary tables**

| Cohort | Mouse number | FASTQ file |
| --- | --- | --- |
|  |  | Raw reads |
| 1 | 1 | 1134791 |
|  | 2 | 2996976 |
|  | 3 | 1887409 |
| 2 | 4 | 15919100 |
|  | 5 | 257022 |
|  | 6 | 2058810 |
| 3 | 9 | 2379843 |
|  | 10 | 6957062 |
|  | 11 | 440220 |
| 4 | 12 | 4801987 |
|  | 13 | 109386 |
|  | 14 | 2135682 |
| 5 | 15 | 499782 |
|  | 16 | 546117 |
|  | 17 | 366319 |
| 6 | 18 | 71265 |
|  | 19 | 4786953 |
|  | 20 | 465928 |

Supplementary Table 1. Raw reads from the FASTQ Illumina file obtained for bone marrow plasma cell samples in this study.

| Gene/genes | Publication | Function | Up- or downregulated in  severe dengue |
| --- | --- | --- | --- |
| S100A12, | (Hoang *et al.*, 2010)^51^ | Inflammation | Upregulated |
| S100P |  |  |  |
| CEACAM6, CEACAM8 | (Hoang *et al.*, 2010)^51^ | Inflammation | Upregulated |
| MS4A3 | (Hoang *et al.*, 2010)^51^ | Hematopoiesis regulation | Upregulated |
| PGLYRP1 | (Hoang *et al.*, 2010)^51^ | Immune cell regulation and Antimicrobial | Upregulated |
| HP | (Hoang *et al.*, 2010)^51^ | Antimicrobial, Inflammation | Upregulated |
| CAMP | (Hoang *et al.*, 2010)^51^ | Antimicrobial | Upregulated |
| MPO | (Hoang *et al.*, 2010)^51^ | Antimicrobial | Upregulated |
| IL1R2 | (Hoang *et al.*, 2010)^51^ | Immune cell regulation | Upregulated |
| GYG | (Robinson *et al.*, 2019)^52^ | Immune cell regulation,  Inflammation | Upregulated |
| CX3CR1 | (Robinson *et al.*, 2019)^52^ | Inflammation | Upregulated |
| MS4A1 | (Zanini *et al.*, 2018)^50^ | B cell development | Upregulated |
| CXCR4 | (Zanini *et al.*, 2018)^50^ | Inflammation | Upregulated |
|  |  |  |  |
| TOR3A | (Zanini *et al.*, 2018)^50^ | Interferon response | Upregulated |
|  |  |  |  |
| DEFA4 | (Robinson *et al.*, 2019)^52^ | Antimicrobial | Upregulated |
| PTPRM | (Robinson *et al.*, 2019)^52^ | Immune cell regulation | Downregulated |
| SPON2 | (Robinson *et al.*, 2019)^52^ | Immune cell regulation | Downregulated |
| GRAP2 | (Robinson *et al.*, 2019)^52^ | Immune cell regulation | Downregulated |
| CACNA2D2, CACNA2D3 | (Robinson *et al.*, 2019)^52^ | Immune cell regulation | Downregulated |
| TMEM63C | (Robinson *et al.*, 2019) | Cation transport | Downregulated |
| AK5 | (Robinson *et al.*, 2019)^52^ | Energetic processes | Downregulated |
| CHD3 | (Robinson *et al.*, 2019)^52^ | Transcription regulation | Downregulated |
| TRERF1 | (Robinson *et al.*, 2019)^52^ | Transcription regulation | Downregulated |
| GBP2 | (Robinson *et al.*, 2019)^52^ | Immune cell regulation and Antimicrobial | Downregulated |
| SERINC5 | (Robinson *et al.*, 2019)^52^ | Amino acid synthesis | Downregulated |
| SOX13 | (Robinson *et al.*, 2019)^52^ | Transcription regulation | Downregulated |
| NCR3 | (Robinson *et al.*, 2019)^52^ | Immune cell regulation | Downregulated |
| ABI3 | (Robinson *et al.*, 2019)^52^ | Actin polymerization | Downregulated |
| C3orf18 | (Robinson *et al.*, 2019)^52^ | Uncharacterized | Downregulated |
| ENPP5 | (Robinson *et al.*, 2019)^52^ | NAD hydrolisis | Downregulated |

Supplementary Table 2. Eukaryotic genes which have been previously reported to be influenced by dengue challenge in expression, function and specification on up- or down-regulation effect.

| p-value | Gene | Cluster |
| --- | --- | --- |
| 2.03*10^-193^ | Fos | 3 |
| 1.68*10^-134^ | Rpl9-ps6 | 3 |
| 5.58*10^-111^ | Rpl10-ps3 | 3 |
| 6.01*10^-102^ | Jun | 3 |
| 4.02*10^-68^ | Jund | 3 |
| 7.69*10^-48^ | Rplp0 | 3 |
| 2.83*10^-47^ | Junb | 3 |
| 1.94*10^-35^ | Rpl35 | 3 |
| 2.35*10^-188^ | Rpl10-ps3 | 7 |
| 4.93*10^-171^ | Rpl9-ps6 | 7 |
| 4.91*10^-129^ | Fos | 7 |
| 5.93*10^-129^ | Jun | 7 |
| 3.81*10^-107^ | Jund | 7 |
| 2.08*10^-103^ | Junb | 7 |
| 3.49*10^-32^ | Rplp0 | 7 |
| 8.07*10^-26^ | Rpl35 | 7 |
| 3.81*10^-114^ | Rpl9-ps6 | 9 |
| 7.33*10^-100^ | Rpl10-ps3 | 9 |
| 5.98*10^-49^ | Jun | 9 |
| 5.99*10^-49^ | Jund | 9 |
| 4.27*10^-48^ | Rplp0 | 9 |
| 2.98*10^-45^ | Fos | 9 |
| 5.09*10^-37^ | Rpl35 | 9 |
| 1.89*10^-29^ | Junb | 9 |
| 8.34*10^-148^ | Rpl9-ps6 | 10 |
| 7.33*10^-147^ | Rpl10-ps3 | 10 |
| 5.86*10^-59^ | Fos | 10 |
| 1.98*10^-41^ | Jun | 10 |
| 5.07*10^-38^ | Jund | 10 |
| 1.70*10^-28^ | Rplp0 | 10 |
| 1.66*10^-27^ | Rpl35 | 10 |
| 4.62*10^-22^ | Junb | 10 |

Supplementary Table 3. Statistical significance of the difference of expression of Ribosomal Protein genes and Transcription factors between control and EDIII. The p-values are calculated for the differentially expressed genes Fos, Rpl9-ps6, Rpl10-ps3, Jun, Jund, Rplp0, Junb, Rpl35 for the clusters 3, 7, 9, 10.
